# Association between osteoporosis under treatment and all-cause and specific-cause mortalities: a nationwide retrospective cohort study in South Korea

**DOI:** 10.1186/s12891-025-08527-w

**Published:** 2025-03-24

**Authors:** Hyun Youk, Hee Young Lee, Eun Young Lee, Yoon Ji Kim, Ji Yeong Park, Hyo Geun Choi, Hyun Sik Kim, Jung Woo Lee

**Affiliations:** 1 Digital Health Laboratory, Wonju College of Medicine, Yonsei University, Wonju, Republic of Korea; 2 Department of Emergency Medicine, Yonsei University Wonju College of Medicine, Wonju, Republic of Korea; 3 Suseoseoulent clinic, Seoul, Republic of Korea; 4 Mdanalytics, Seoul, Republic of Korea; 5 Wonju College of Medicine, Yonsei University, Wonju, Republic of Korea; 6 Department of Orthopedic Surgery, Yonsei University Wonju College of Medicine, Wonju, Republic of Korea; 7 Yonsei Institute of Sports Science and Exercise Medicine, Wonju, Republic of Korea; 8 Biobytes, Co., Ltd, Wonju, Republic of Korea

**Keywords:** Osteoporosis, Treatment, Mortality, Retrospective cohort, South Korea

## Abstract

**Background:**

Despite the association between osteoporosis treatment and reduced mortality, evidence on specific-cause mortality is lacking. Therefore, this study explored the association between osteoporosis under treatment and all-cause and specific-cause mortalities using nationwide retrospective cohort data from South Korea.

**Methods:**

This study utilized data from the National Health Insurance Service screening cohort of South Korea from 2002 to 2019. Participants with osteoporosis who had undergone treatment at least twice and were diagnosed based on bone densitometry were included. Control groups were matched 1:1 based on age, sex, income, and region. Propensity score overlap weighting was applied to balance covariates. Cox proportional hazards models and Fine–Gray sub-distribution hazard models were used to assess all-cause and specific-cause mortalities across 14 disease categories based on the Korean standard classification of diseases.

**Results:**

Finally, 34,181 participants were included in both osteoporosis and control groups. The largest age group was 55–59 years, with a majority of female participants (81.60%). Osteoporosis under treatment was significantly associated with reduced all-cause mortality with consistent results across various demographic and clinical subgroups. Specific-cause mortality analysis revealed lower mortality due to neoplasms and metabolic diseases and higher mortality from respiratory and muscular diseases. However, increased risks of respiratory and muscular disease-related mortality were observed.

**Conclusions:**

Osteoporosis treatment was associated with reduced all-cause and specific-cause mortalities, particularly from neoplasms and metabolic diseases. Further studies, particularly randomized controlled trials, are required to confirm these results, establish causality, and explore the medication-specific effects on mortality.

## Background

Osteoporosis is a systemic skeletal disease characterized by reduced bone mineral density and deterioration of bone microarchitecture, leading to increased bone fragility and a heightened risk of fractures. According to the Global Burden of Disease 2019 study for low bone mineral density that included both osteoporosis and low bone mass, the age-standardized disability-adjusted life-years and mortality rate were estimated to be 206.85 (95% uncertainty interval [UI], 167.92–248.69) and 5.74 (95% UI, 4.72–6.51) per 100,000 individuals in 2019, respectively [1]. According to a recent systematic review, the prevalence of osteoporosis was found to be 10–30% in older women and up to 10% in men in the Asia-Pacific region [2]. These statistics highlighted the significant burden of osteoporosis, which is particularly pronounced among older adults.

Osteoporosis leads to a substantial decrease in bone strength, increasing the risk of fractures even with minimal trauma [3]. Chandran et al. reported that the incidence of osteoporotic fractures among individuals aged ≥ 50 years ranged from 500 to 1,000 per 100,000 person-years in the Asia-Pacific region [2]. Osteoporotic fractures are associated with significant bone-related morbidities, elevated mortality rates, and higher healthcare costs [3]. Although hip and vertebral fractures are not the most frequent osteoporosis-related fractures, they are the most severe, leading to substantially increased morbidity and mortality [4–6]. A retrospective cohort study with approximately 4,000 participants found that the annual mortality rate following osteoporotic hip fractures was 144.9 per 1,000 patients per year. The study also observed a yearly increase in the 1-year mortality rate by 2% (incidence rate ratio 1.020, 95% confidence interval [CI] 1.008–1.033) [7]. Although the risk of mortality is moderated by numerous factors including age, sex, and comorbid conditions, fractures related to osteoporosis were found to be directly responsible for a portion of the increased mortality [8]. Numerous previous studies have investigated the association between osteoporosis treatment and mortality [9–13] and reported reduced mortality rates in the treatment group than in the control group. However, although these previous studies were well-conducted, they primarily focused on reporting all-cause mortality. Some studies did investigate specific causes of death, but they examined only a few diseases of their interest and did not employ a systematic approach.

Herein, we investigated the association between osteoporosis under treatment and all-cause and specific-cause mortalities using nationwide retrospective cohort data in South Korea. Notably, we explored the specific-cause mortality for all 14 categories according to the Korean standard classification of diseases (KCD), which is based on the International Classification of Diseases (ICD) developed by the World Health Organization. By delving into the specific causes of death related to osteoporosis, our study aimed to provide a more nuanced understanding of the association of osteoporosis treatments with mortality beyond conventional approaches. Understanding the specific causes of death associated with osteoporosis could provide further insights regarding underlying mechanisms into the well-established observational association between osteoporosis treatment and reduced mortality.

## Methods

### Study ethics

This study was approved by the ethics committee of Hallym University and was conducted in accordance with its guidelines and regulations. The requirement for written informed consent was waived by the Institutional Review Board because this study utilized retrospective and de-identified data (registration number: 2019-10-023).

### Participant recruitment

We utilised the data of the National Health Insurance Service (NHIS) screening cohort of South Korea from 2002 to 2019. A detailed explanation regarding the NHIS screening cohort is described elsewhere [14]. Participants with osteoporosis were selected from 514,866 participants with 895,300,177 medical claim codes recorded from 2002 to 2019 (*n* = 117,946). Participants with a diagnosis record of osteoporosis in 2002 were excluded to include only the newly diagnosed cases of osteoporosis (washout periods, *n* = 15,510). For analysis, we excluded the participants with missing data: participants with osteoporosis who had no records of fasting blood glucose (FBG; *n* = 8), body mass index (BMI; *n* = 7), total cholesterol (*n* = 5), and blood pressure (*n* = 1) were excluded. Thus, a total of 15,531 participants were excluded before matching. The participants for the control group were included if they were not defined as having osteoporosis from 2002 to 2019 (*n* = 396,920). In the control group, participants who have never been diagnosed with osteoporosis were included (*n* = 66,089).

**Fig. 1:**
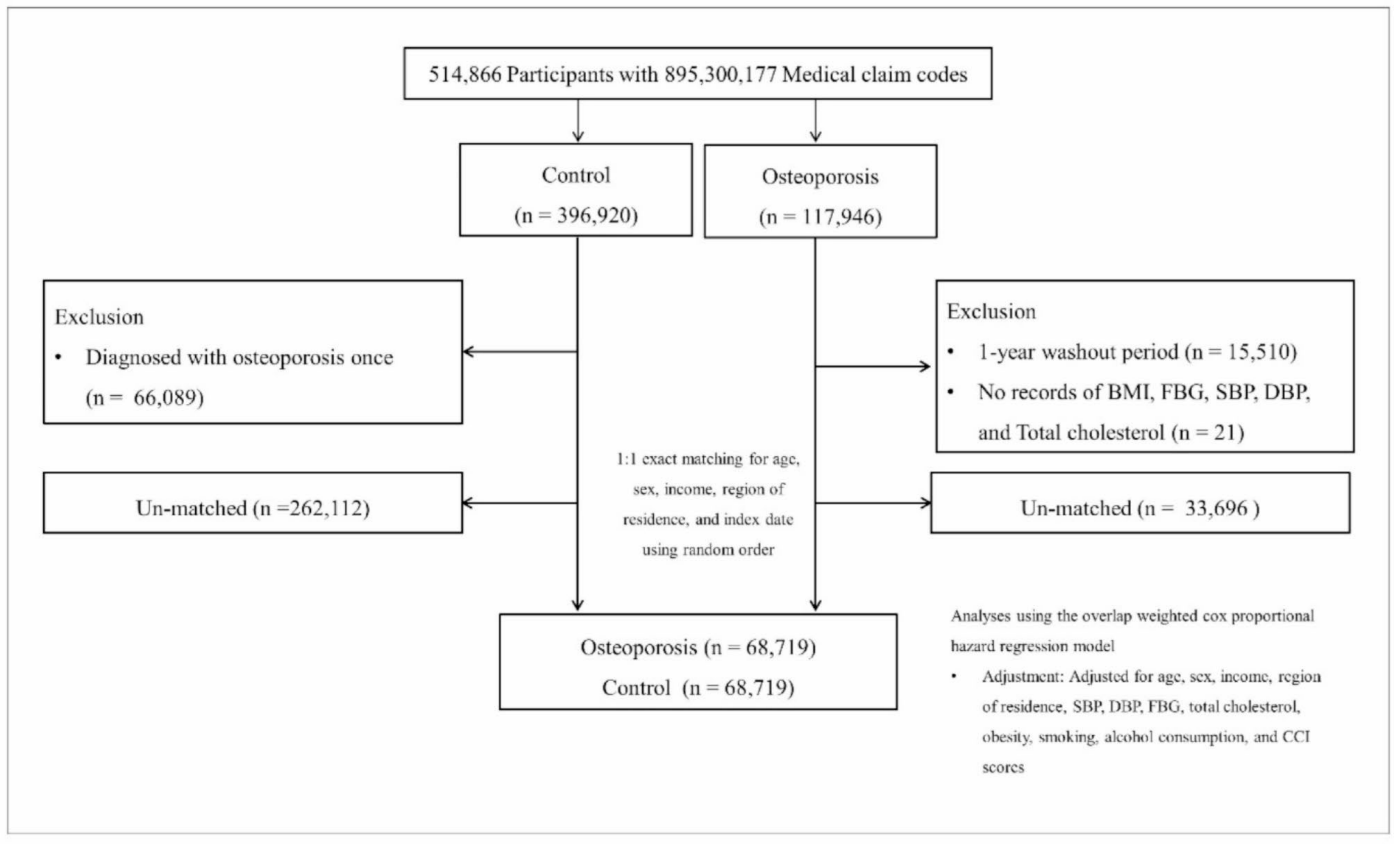
A schematic illustration of the participant selection process that was used in the present study. Of a total of 514,866 participants, 68,719 of osteoporosis participants were 1:1 matched with 68,719 of control participants for age, sex, income, and region of residence

Participants in the osteoporosis group were matched 1:1 with those in the control group based on age, sex, income, and region of residence. To prevent selection bias in the selection process of the matched participants, the control-group participants were first randomised by assigning a random number order and then selected sequentially from the top of the list. The matched control participants were assumed to be evaluated simultaneously as each matched participant with osteoporosis on the index date. Therefore, the control-group participants who died before the index date were excluded (left-truncated). During the matching procedure, 262,112 and 33,696 participants in the control and osteoporosis groups, respectively, were excluded. Finally, 68,719 participants with osteoporosis were 1:1 matched with those in the control group (Fig. 1).

### Exposure: osteoporosis

Osteoporosis was defined as a diagnosis of ICD (10th edition) codes M80 (osteoporosis with pathological fracture) or M81 (osteoporosis without pathological fracture) occurring from 2002 to 2019. Among them, we selected the participants who had undergone treatment for osteoporosis at least twice and the participants who were diagnosed with osteoporosis by bone densitometry using dual-energy X-ray absorptiometry (DEXA) or DEXA computed tomography (claim code: E7001-E7004).

### Outcomes: all-cause and specific-cause mortalities

The primary outcome was all-cause mortality and the secondary outcome was specific-cause mortality. The specific-cause mortality was classified into 14 categories according to the KCD: (1) infections and parasitic diseases (A00–B99); (2) neoplasms (C00–D48); (3) metabolic diseases (E00–E90); (4) mental and behavioral disorders (F00–F99); (5) neurologic diseases (G00–G99); (6) circulatory diseases (I00–I99); (7) respiratory diseases (J00–J99); (8) digestive diseases (K00–K93); (9) muscular disease (M00–M99); (10) diseases of the musculoskeletal system and connective tissue (M00–M99); (11) genitourinary diseases (N00–N99); (12) abnormal finding (not elsewhere classified, R00–R99); (13) trauma (injury, poisoning, and certain other consequences of external causes, S00–T98); and (14) others (diseases of the blood and blood-forming organs and certain immune system disorders, D50–D89; diseases of the skin and subcutaneous tissue, L00–L99; diseases of the eye and adnexa, H00–H59; diseases of the ear and mastoid process, H60–H95; pregnancy, childbirth and the puerperium, O00–O99; and congenital malformations, deformations, and chromosomal abnormalities, Q00–Q89).

### Covariates

Participants were stratified into 10 age groups at 5-year intervals, starting from 40 to 44 years up to ≥ 85 years. Income levels were divided into five categories, from class 1 (lowest income) to class 5 (highest income). Residential areas were classified as either urban or rural, according to the methodology used in our previous study [15]. Smoking status was divided into two categories (non-smoker and past and current smoker). Alcohol consumption was classified into two categories: less than once per week and once or more per week, irrespective of the amount consumed per occasion. BMI was categorized into four groups: underweight (< 18.5 kg/m^2^), normal (≥ 18.5 to < 23 kg/m^2^), overweight (≥ 23 to < 25 kg/m^2^), and obese (≥ 25 kg/m^2^). Records of systolic blood pressure (SBP, mmHg), diastolic blood pressure (DBP, mmHg), FBG (mg/dL), and total cholesterol (mg/dL) were utilized. The Charlson Comorbidity Index (CCI) that measures disease burden based on 17 comorbidities was used. Each participant was assigned a score based on the severity and number of diseases, with the CCI measured as a continuous variable ranging from 0 (no comorbidities) to 29 (multiple comorbidities).

### Statistical analyses

We applied propensity score (PS) overlap weighting to ensure covariate balance and optimize the effective sample size. The PS was determined using multivariable logistic regression, incorporating all covariates. To calculate overlap weighting, participants in the osteoporosis group were weighted by their PS, whereas those in the control group were weighted by ‘1 – PS’. This method generated weights ranging from 0 to 1, achieving exact balance and enhancing statistical precision [16–18]. The standardized difference, both before and after weighting, was used to compare the general characteristics between the osteoporosis and control groups.

To analyze the overlap-weighted hazard ratios (HRs) of osteoporosis for all-cause mortality, we employed the PS overlap-weighted Cox cause-specific proportional hazards model. This analysis included both crude (unadjusted) and overlap-weighted models, with adjustments made for age, sex, income, region of residence, SBP, DBP, FBG, total cholesterol, BMI, smoking status, alcohol consumption, and CCI scores. We also performed subgroup analyses for all-cause mortality, examining variations based on age, sex, income, region of residence, BMI, smoking status, alcohol consumption status, blood pressure, FBG, total cholesterol, and CCI scores.

Crude and overlap-weighted sub-distribution proportional hazards model of osteoporosis for mortality according to individual cause of death were analyzed (cause-specific mortality). The Fine–Gray sub-distribution hazards model was employed to estimate subject-specific probabilities of the event of interest over time while accounting for competing risks [19]. In this study, mortality due to other causes was regarded as a competing risk. We calculated 95% CIs and incidence rate differences (IRDs). Kaplan–Meier survival analysis and the log-rank test were also used. Statistical significance was determined at a threshold of *P* < 0.05, and all analyses were conducted with two-sided tests. SAS version 9.4 (SAS Institute Inc., Cary, NC, USA) was used for all statistical analyses.

## Results

### Participants’ characteristics

After the PS overlap-weighting adjustment, 34,181 participants were included in both the osteoporosis and the matched control groups. The age group of 55–59 years (22.55%) and women (81.60%) constituted the majority of the participants. There were 4,039 (11.82%) and 5,911 (17.29%) mortality cases in the osteoporosis and control groups, respectively, with a standardized difference of 0.16. Detailed information on participants’ characteristics in this study is presented in Table 1.

**Table 1:** General characteristics of participants propensity score overlap weighting adjustment

Characteristics	After PS Overlap weighting adjustment			Before PS Overlap weighting adjustment		
	Osteoporosis (n, %)	Control (n, %)	Standardized Difference	Osteoporosis (n, %)	Control (n, %)	Standardized Difference
Total participants (n, %)						
Age (%)			0.00			0.00
40–44	505 (1.48)	505 (1.48)		1,021 (1.47)	1,021 (1.47)	
45–49	2,533 (7.41)	2,533 (7.41)		5,139 (7.40)	5,139 (7.40)	
50–54	6,023 (17.62)	6,023 (17.62)		12,243 (17.64)	12,243 (17.64)	
55–59	7,707 (22.55)	7,707 (22.55)		15,683 (22.59)	15,683 (22.59)	
60–64	6,882 (20.13)	6,882 (20.13)		13,961 (20.11)	13,961 (20.11)	
65–69	3,654 (10.69)	3,654 (10.69)		7,430 (10.70)	7,430 (10.70)	
70–74	3,509 (10.27)	3,509 (10.27)		7,109 (10.24)	7,109 (10.24)	
75–79	2,252 (6.59)	2,252 (6.59)		4,569 (6.58)	4,569 (6.58)	
80–84	929 (2.72)	929 (2.72)		1,882 (2.71)	1,882 (2.71)	
85+	187 (0.55)	187 (0.55)		381 (0.55)	381 (0.55)	
Sex (%)			0.00			0.00
Male	6,291 (18.40)	6,291 (18.40)		12,801 (18.44)	12,801 (18.44)	
Female	27,890 (81.60)	27,890 (81.60)		56,617 (81.56)	56,617 (81.56)	
Income (%)			0.00			0.00
1 (lowest)	6,466 (18.92)	6,466 (18.92)		13,123 (18.90)	13,123 (18.90)	
2	5,192 (15.19)	5,192 (15.19)		10,538 (15.18)	10,538 (15.18)	
3	5,602 (16.39)	5,602 (16.39)		11,392 (16.41)	11,392 (16.41)	
4	6,953 (20.34)	6,953 (20.34)		14,138 (20.37)	14,138 (20.37)	
5 (highest)	9,968 (29.16)	9,968 (29.16)		20,227 (29.14)	20,227 (29.14)	
Region of residence (%)			0.00			0.00
Urban	14,458 (42.30)	14,458 (42.30)		29,337 (42.26)	29,337 (42.26)	
Rural	19,723 (57.70)	19,723 (57.70)		40,081 (57.74)	40,081 (57.74)	
Obesity † (%)			0.00			0.18
Underweight	989 (2.89)	989 (2.89)		2,449 (3.53)	1,683 (2.42)	
Normal	12,728 (37.24)	12,728 (37.24)		27,940 (40.25)	23,702 (34.14)	
Overweight	9,074 (26.55)	9,074 (26.55)		18,110 (26.09)	18,416 (26.53)	
Obese I	10,258 (30.01)	10,258 (30.01)		19,080 (27.49)	22,607 (32.57)	
Obese II	1,132 (3.31)	1,132 (3.31)		1,839 (2.65)	3,010 (4.34)	
Smoking status (%)			0.00			0.04
Nonsmoker	30,049 (87.91)	30,049 (87.91)		61,316 (88.33)	60,663 (87.39)	
Past smoker	1,839 (5.38)	1,839 (5.38)		3,731 (5.37)	3,733 (5.38)	
Current smoker	2,293 (6.71)	2,293 (6.71)		4,371 (6.30)	5,022 (7.23)	
Alcohol consumption (%)			0.00			0.03
< 1 time a week	27,991 (81.89)	27,991 (81.89)		57,210 (82.41)	56,457 (81.33)	
≥ 1 time a week	6,190 (18.11)	6,190 (18.11)		12,208 (17.59)	12,961 (18.67)	
SBP (Mean, SD)	126.51 (12.48)	126.51 (12.79)	0.00	125.44 (17.58)	127.72 (18.56)	0.13
DBP (Mean, SD)	77.82 (7.75)	77.82 (7.91)	0.00	77.25 (10.95)	78.46 (11.40)	0.11
FBG (Mean, SD)	98.57 (22.29)	98.57 (19.12)	0.00	97.01 (27.15)	100.93 (33.49)	0.13
Total cholesterol (Mean, SD)	202.98 (27.49)	202.98 (27.53)	0.00	202.42 (38.93)	203.66 (39.50)	0.03
CCI score (Mean, SD)	1.07 (1.18)	1.07 (1.27)	0.00	1.10 (1.73)	1.04 (1.77)	0.03
Mortality (n, %)	4,039 (11.82)	5,911 (17.29)	0.16	8,356 (12.04)	11,858 (17.08)	0.14

Abbreviations: CCI, Charlson Comorbidity Index; SD, Standard deviation; SBP, Systolic blood pressure; DBP, Diastolic blood pressure; FBG, Fasting blood glucose; PS, Propensity score

† Obesity (BMI, body mass index, kg/m^2^) was categorized as < 18.5 (underweight), ≥ 18.5 to < 23 (normal), ≥ 23 to < 25 (overweight), ≥ 25 to < 30 (obese I), and ≥ 30 (obese II)

### Association between osteoporosis under treatment and all-cause mortality

In the main analysis using data from all participants, the crude model indicated that osteoporosis under treatment was associated with a reduced likelihood of all-cause mortality (HR 0.66, 95% CI 0.65–0.68, *P* < 0.001). This association remained significant in the overlap-weighted model, although the effect size was slightly amplified (HR 0.59, 95% CI 0.57–0.60, *P* < 0.001; Table 2). The IRD for the association between osteoporosis under treatment and all-cause mortality was estimated to be − 5.70 (95% CI − 6.09 to − 5.31; Table 2).

**Table 2:** Crude and overlap propensity score weighted hazard ratios (95% confidence interval) of osteoporosis for mortality with subgroup analyses

	*N* of event / *N* of total (%)	F/U duration (PY)	IR per 1000 (PY)	IRD (95% CI)	Hazard ratios (95% confidence interval)			
					Crude	*P*-value	Overlap weighted model †	*P*-value
**Total participants**								
Osteoporosis	8,356 / 69,418 (12.04)	736,225	11.30	-5.70 (-6.09–5.31)	0.66 (0.65–0.68)	< 0.001*	0.59 (0.57–0.6)	< 0.001*
Control	11,858 / 69,418 (17.08)	695,621	17.00		1		1	
**Age < 60 years old**								
Osteoporosis	716 / 34,086 (2.10)	385,472	1.86	-1.98 (-2.22–1.74)	0.48(0.44–0.53)	< 0.001*	0.46(0.42–0.50)	< 0.001*
Control	1,454 / 34,086 (4.27)	379,120	3.84		1		1	
**Age ≥ 60 years old**								
Osteoporosis	7,640 / 35,332 (21.62)	350,753	21.80	-11.10 (-11.88–10.30)	0.66(0.64–0.68)	< 0.001*	0.60(0.59–0.62)	< 0.001*
Control	10,404 / 35,332 (29.45)	316,501	32.90		1		1	
**Male**								
Osteoporosis	3,235 / 12,801 (25.27)	92,418	35.00	-5.90 (-7.67–4.07)	0.86(0.82–0.90)	< 0.001*	0.75(0.71–0.78)	< 0.001*
Control	3,579 / 12,801 (27.96)	87,567	40.90		1		1	
**Female**								
Osteoporosis	5,121 / 56,617 (9.04)	643,807	7.95	-5.65 (-6.02–5.30)	0.58(0.56–0.60)	< 0.001*	0.52(0.50–0.54)	< 0.001*
Control	8,279 / 56,617 (14.62)	608,054	13.60		1		1	
**Low income**								
Osteoporosis	4,450 / 35,053 (12.70)	370,419	12.00	-6.30 (-6.87–5.73)	0.65(0.63–0.68)	< 0.001*	0.58(0.55–0.60)	< 0.001*
Control	6,372 / 35,053 (18.18)	347,898	18.30		1		1	
**High income**								
Osteoporosis	3,906 / 34,365 (11.37)	365,806	10.70	-5.10 (-5.63–4.57)	0.68(0.65–0.70)	< 0.001*	0.60(0.57–0.62)	< 0.001*
Control	5,486 / 34,365 (15.96)	347,723	15.80		1		1	
**Urban resident**								
Osteoporosis	2,752 / 29,337 (9.38)	314,231	8.76	-4.24 (-4.75–3.71)	0.67(0.64–0.71)	< 0.001*	0.58(0.55–0.61)	< 0.001*
Control	3,913 / 29,337 (13.34)	301,216	13.00		1		1	
**Rural resident**								
Osteoporosis	5,604 / 40,081 (13.98)	421,994	13.30	-6.80 (-7.42–6.31)	0.66(0.64–0.68)	< 0.001*	0.59(0.57–0.61)	< 0.001*
Control	7,945 / 40,081 (19.82)	394,405	20.10		1		1	
**Underweight**								
Osteoporosis	621 / 2,449 (25.36)	21,910	28.30	-18.60 (-22.58–14.55)	0.61(0.54–0.68)	< 0.001*	0.68(0.61–0.76)	< 0.001*
Control	647 / 1,683 (38.44)	13,794	46.90		1		1	
**Normal weight**								
Osteoporosis	3,527 / 27,940 (12.62)	287,187	12.30	-7.10 (-7.84–6.48)	0.63(0.60–0.66)	< 0.001*	0.60(0.57–0.62)	< 0.001*
Control	4,456 / 23,702 (18.80)	229,197	19.40		1		1	
**Overweight**								
Osteoporosis	1,868 / 18,110 (10.31)	195,323	9.56	-5.64 (-6.37–4.96)	0.62(0.59–0.66)	< 0.001*	0.56(0.52–0.59)	< 0.001*
Control	2,815 / 18,416 (15.29)	184,847	15.20		1		1	
**Obese**								
Osteoporosis	2,340 / 20,919 (11.19)	231,805	10.10	-4.60 (-5.24–4.00)	0.68(0.65–0.72)	< 0.001*	0.58(0.55–0.61)	< 0.001*
Control	3,940 / 25,617 (15.38)	267,783	14.70		1		1	
**Non-smoker**								
Osteoporosis	6,456 / 61,316 (10.53)	672,765	9.60	-5.30 (-5.69–4.93)	0.64(0.62–0.66)	< 0.001*	0.56(0.54–0.58)	< 0.001*
Control	9,395 / 60,663 (15.49)	630,435	14.90		1		1	
**Past smoker and current smoker**								
Osteoporosis	1,900 / 8,102 (23.45)	63,460	29.90	-7.90 (-9.86–5.83)	0.79(0.75–0.84)	< 0.001*	0.69(0.65–0.73)	< 0.001*
Control	2,463 / 8,755 (28.13)	65,186	37.80		1		1	
**Alcohol consumption < 1 time a week**								
Osteoporosis	6,888 / 57,210 (12.04)	647,875	10.60	-6.10 (-6.48–5.66)	0.63(0.62–0.65)	< 0.001*	0.56(0.55–0.58)	< 0.001*
Control	10,072 / 56,457 (17.84)	603,019	16.70		1		1	
**Alcohol consumption ≥ 1 time a week**								
Osteoporosis	1,468 / 12,208 (12.02)	88,350	16.60	-2.70 (-3.91–1.44)	0.86(0.81–0.93)	< 0.001*	0.72(0.67–0.77)	< 0.001*
Control	1,786 / 12,961 (13.78)	92,602	19.30		1		1	
**SBP < 140 mmHg and DBP < 90 mmHg**								
Osteoporosis	5,035 / 51,649 (9.75)	533,336	9.44	-3.96 (-4.38–3.55)	0.70(0.68–0.73)	< 0.001*	0.60(0.58–0.62)	< 0.001*
Control	6,364 / 48,460 (13.13)	474,723	13.40		1		1	
**SBP ≥ 140 mmHg or DBP ≥ 90 mmHg**								
Osteoporosis	3,321 / 17,769 (18.69)	202,889	16.40	-8.50 (-9.37–7.63)	0.66(0.63–0.69)	< 0.001*	0.57(0.54–0.59)	< 0.001*
Control	5,494 / 20,958 (26.21)	220,898	24.90		1		1	
**FBG < 100 mg/dL**								
Osteoporosis	5,139 / 48,301 (10.64)	524,271	9.80	-4.10 (-4.50–3.65)	0.70(0.68–0.73)	< 0.001*	0.59(0.57–0.61)	< 0.001*
Control	6,452 / 44,850 (14.39)	464,975	13.90		1		1	
**FBG ≥ 100 mg/dL**								
Osteoporosis	3,217 / 21,117 (15.23)	211,954	15.20	-8.20 (-9.08–7.44)	0.65(0.62–0.67)	< 0.001*	0.58(0.56–0.61)	< 0.001*
Control	5,406 / 24,568 (22.00)	230,646	23.40		1		1	
**Total cholesterol < 200 mg/dL**								
Osteoporosis	4,420 / 34,327 (12.88)	353,644	12.50	-6.30 (-6.92–5.73)	0.66(0.64–0.69)	< 0.001*	0.59(0.57–0.61)	< 0.001*
Control	6,105 / 33,437 (18.26)	324,296	18.80		1		1	
**Total cholesterol ≥ 200 mg/dL**								
Osteoporosis	3,936 / 35,091 (11.22)	382,581	10.30	-5.20 (-5.72–4.69)	0.66(0.63–0.69)	< 0.001*	0.58(0.56–0.60)	< 0.001*
Control	5,753 / 35,981 (15.99)	371,325	15.50		1		1	
**CCI scores = 0**								
Osteoporosis	1,036 / 38,140 (2.72)	407,274	2.54	-1.97 (-2.22–1.71)	0.57(0.52–0.61)	< 0.001*	0.57(0.53–0.62)	< 0.001*
Control	1,954 / 41,630 (4.69)	433,437	4.51		1		1	
**CCI scores = 1**								
Osteoporosis	1,504 / 12,848 (11.71)	140,228	10.70	-7.40 (-8.31–6.45)	0.59(0.55–0.63)	< 0.001*	0.59(0.55–0.63)	< 0.001*
Control	1,996 / 10,845 (18.40)	110,249	18.10		1		1	
**CCI scores ≥ 2**								
Osteoporosis	5,816 / 18,430 (31.56)	188,723	30.80	-21.20 (-22.59–19.87)	0.58(0.57–0.60)	< 0.001*	0.57(0.55–0.59)	< 0.001*
Control	7,908 / 16,943 (46.67)	151,935	52.00		1		1	

Abbreviation: CI, Confidence Interval; CCI, Charlson Comorbidity Index; IR, incidence rate; IRD, incidence rate difference; SBP, systolic blood pressure; DBP, diastolic blood pressure; FBG. Fasting blood glucose; PY. person-year;

* Significance at *P* < 0.05

† Adjusted for age, sex, income, region of residence, SBP, DBP, fasting blood glucose, total cholesterol, obesity, smoking, alcohol consumption, and CCI scores

**Fig. 2:**
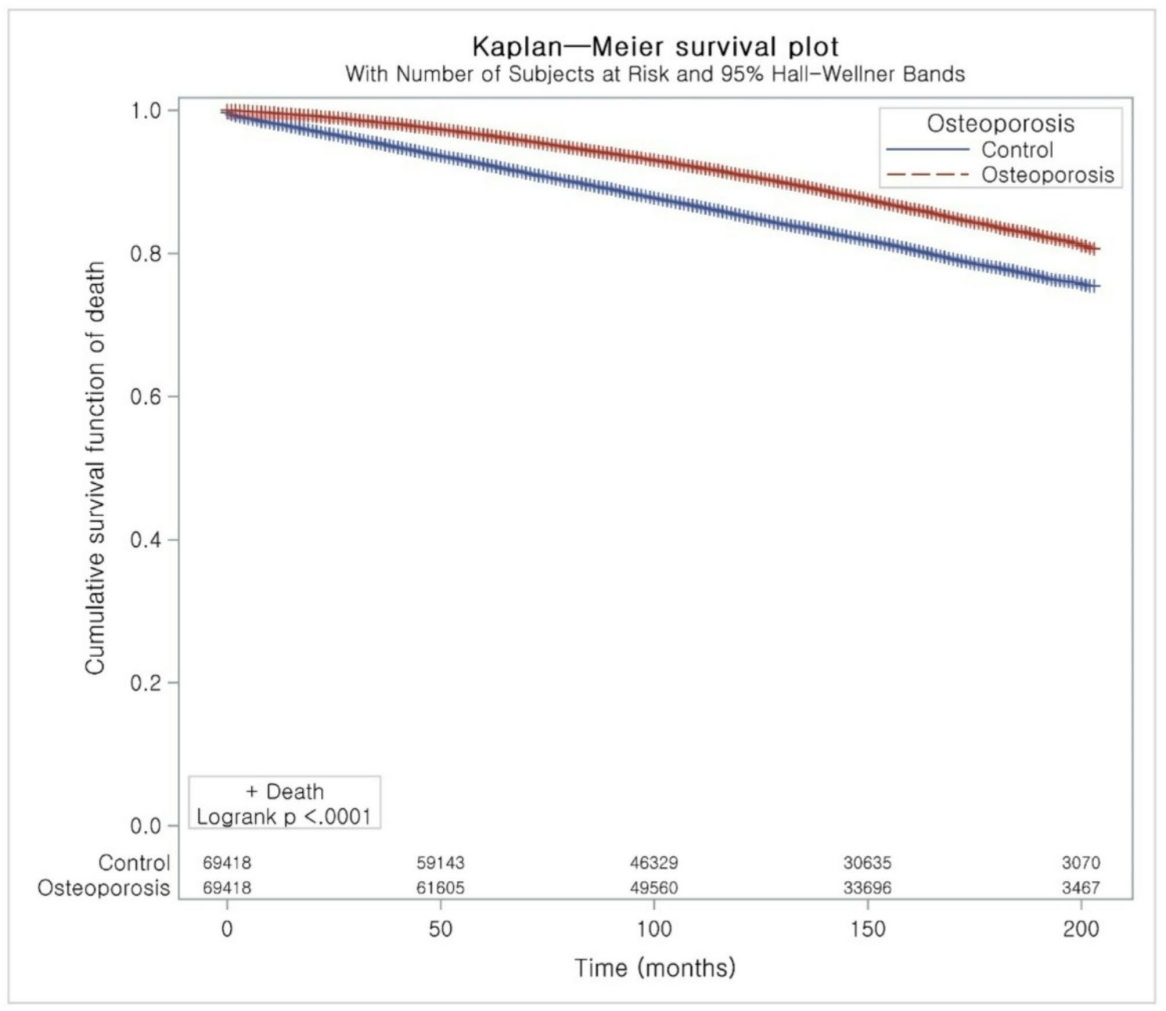
Kaplan-Meier survival plot showing the cumulative survival function of death for osteoporosis (dashed red line) and control (solid blue line)

**Fig. 3:**
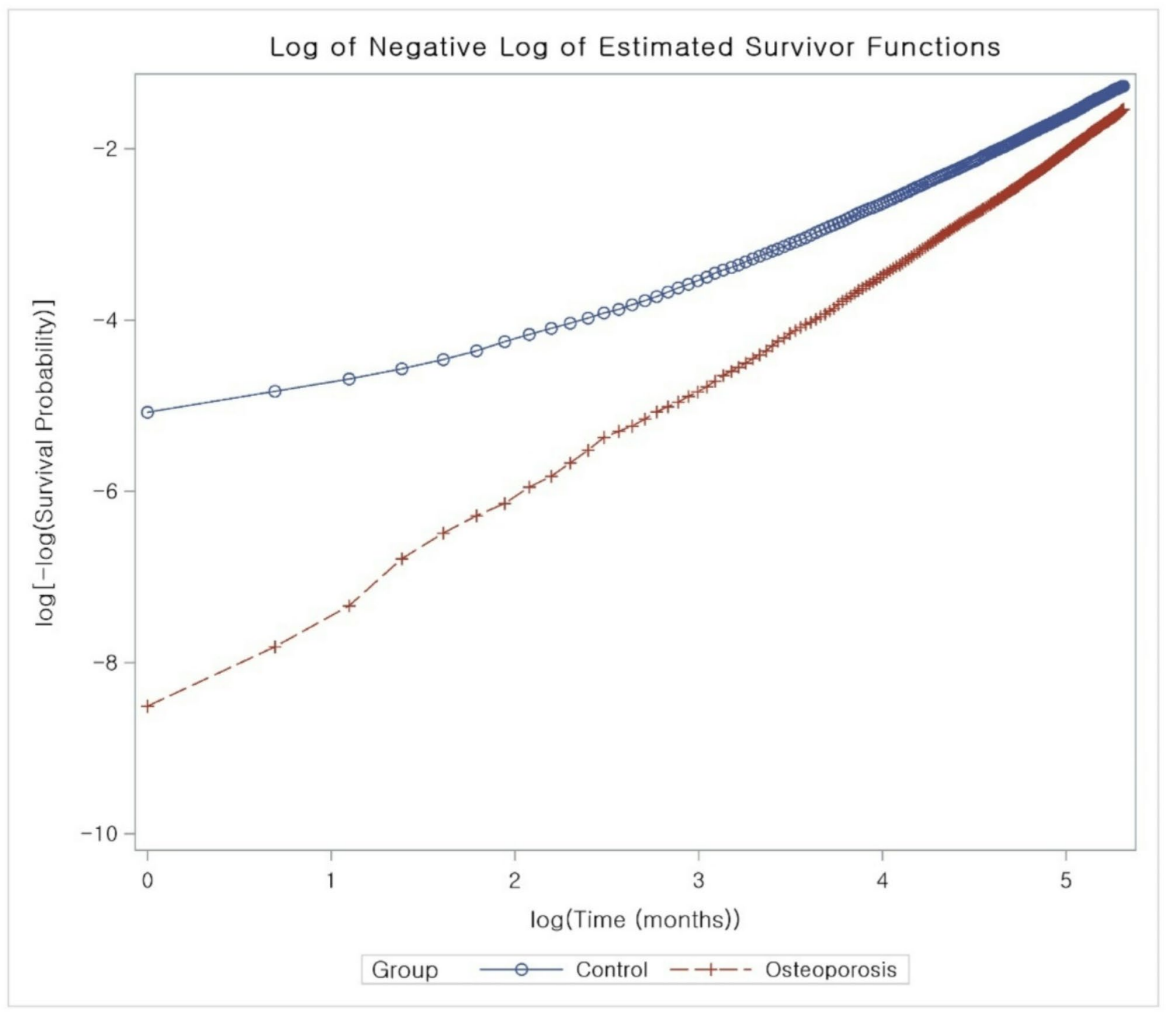
Log-log survival plot displaying the log of negative log of estimated survivor functions for osteoporosis (dashed red line with cross markers) and control (solid blue line with circular markers)

Figure 2 illustrates the Kaplan–Meier survival curves for osteoporosis and control groups, which suggested a statistically significantly lower mortality rate for the osteoporosis group over the follow-up period (*P* < 0.0001). Figure 3 presents the log-negative-log of estimated survivor functions, showing a distinct divergence with the osteoporosis group consistently demonstrating a higher survival probability. Both of these analyses indicated that participants with osteoporosis under treatment were associated with a reduced all-cause mortality rate than those in the control group.

### Subgroup analyses for all-cause mortality

Our subgroup analyses showed that osteoporosis under treatment was consistently associated with a reduced risk of all-cause mortality across investigated demographics and clinical characteristics (Table 2). Specifically, participants aged < 60 years (HR 0.46, 95% CI 0.42–0.50) and those aged ≥ 60 years (HR 0.60, 95% CI 0.59–0.62) both showed significantly lower mortality rates than their control counterparts. Women (HR 0.52, 95% CI 0.50–0.54) were associated with a more pronounced decrease in all-cause mortality than men (HR 0.75, 95% CI 0.71–0.78).

Regarding income levels, both low-income (HR 0.58, 95% CI 0.55–0.60) and high-income (HR 0.60, 95% CI 0.57–0.62) groups showed similar effect sizes. Similarly, for regions of residence, participants living in urban (HR 0.58, 95% CI 0.55–0.61) and rural (HR 0.59, 95% CI 0.57–0.61) areas were associated with reduced all-cause mortality, exhibiting comparable effect sizes. For BMI, participants with underweight (HR 0.68, 95% CI 0.61–0.76), normal weight (HR 0.60, 95% CI 0.57–0.62), overweight (HR 0.56, 95% CI 0.52–0.59), and obesity (HR 0.58, 95% CI 0.55–0.61) showed a significant association with reduced all-cause mortality. In terms of smoking status, non-smokers (HR 0.56, 95% CI 0.54–0.58) and past and current smokers (HR 0.69, 95% CI 0.65–0.73) showed an association with reduced mortality risk. Regarding alcohol consumption, participants consuming alcohol less than once a week (HR 0.56, 95% CI 0.55–0.58) and those consuming alcohol more frequently (HR 0.72, 95% CI 0.67–0.77) showed an association with reduced mortality risk.

Participants with normal (SBP < 140 mmHg and DBP < 90 mmHg; HR 0.60, 95% CI 0.58–0.62) and elevated (SBP ≥ 140 mmHg or DBP ≥ 90 mmHg; HR 0.57, 95% CI 0.54–0.59) blood pressures had an associated lower mortality risk. Participants with normal (< 100 mg/dL; HR 0.59, 95% CI 0.57–0.61) and higher blood glucose levels (≥ 100 mg/dL; HR 0.58, 95% CI 0.56–0.61) had an associated lower mortality risk. Participants with total cholesterol levels below 200 mg/dL (HR 0.59, 95% CI 0.57–0.61) and those with levels of ≥ 200 mg/dL (HR 0.58, 95% CI 0.56–0.60) exhibited associations with reduced all-cause mortality. Participants with no comorbidities (CCI score = 0; HR 0.57, 95% CI 0.53–0.62) and those with a CCI score of 1 (HR 0.59, 95% CI 0.55–0.63) and ≥ 2 (HR 0.57, 95% CI 0.55–0.59) showed associations with reduced mortality risks.

### Association between osteoporosis under treatment and specific-cause mortality

Our analysis of the association between osteoporosis under treatment and specific-cause mortality revealed that neoplasm-related deaths (HR 0.74, 95% CI 0.69–0.80, *P* < 0.001) and metabolic disease-related mortality (HR 0.71, 95% CI 0.57–0.89, *P* = 0.003) were significantly lower in the osteoporosis group. However, respiratory disease-related mortality (HR 1.35, 95% CI 1.19–1.53, *P* < 0.001) and muscular disease-related mortality (HR 5.38, 95% CI 3.01–9.61, *P* < 0.001; Table 3) were significantly higher in the osteoporosis group.

**Table 3:** Subdistribution hazard model for competing risk analysis in mortality between osteoporosis and control group according to individual cause of death

Cause of death	N of event / N of total (%)	IR per 1000 (PY)	IRD (95% CI)	Subdistribution hazard ratios (95% confidence interval)			
				Crude	P-value	Overlap weighted model †	P-value
**Infection**							
Osteoporosis	247 / 69,418 (0.36)	0.34	-0.09 (-0.16–0.03)	1.20 (0.94–1.53)	0.137	1.19 (0.93–1.52)	0.161
Control	297 / 69,418 (0.43)	0.43		1		1	
**Neoplasm**							
Osteoporosis	2,384 / 69,418 (3.43)	3.24	-2.26 (-2.47–2.04)	0.75 (0.70–0.81)	< 0.001*	0.74 (0.69–0.80)	< 0.001*
Control	3,824 / 69,418 (5.51)	5.50		1		1	
**Metabolic disease**							
Osteoporosis	272 / 69,418 (0.39)	0.37	-0.50 (-0.59–0.42)	0.76 (0.62–0.94)	0.009*	0.71 (0.57–0.89)	0.003*
Control	608 / 69,418 (0.88)	0.87		1		1	
**Mental disease**							
Osteoporosis	126 / 69,418 (0.18)	0.17	-0.10 (-0.15–0.05)	0.97 (0.70–1.33)	0.839	0.93 (0.67–1.28)	0.636
Control	188 / 69,418 (0.27)	0.27		1		1	
**Neurologic disease**							
Osteoporosis	354 / 69,418 (0.51)	0.48	-0.15 (-0.23–0.08)	1.13 (0.92–1.37)	0.245	1.10 (0.90–1.35)	0.342
Control	441 / 69,418 (0.64)	0.63		1		1	
**Circulatory disease**							
Osteoporosis	1,923 / 69,418 (2.77)	2.61	-1.54 (-1.73–1.35)	0.96 (0.88–1.04)	0.301	0.93 (0.85–1.01)	0.079
Control	2,887 / 69,418 (4.16)	4.15		1		1	
**Respiratory disease**							
Osteoporosis	1,065 / 69,418 (1.53)	1.45	-0.04 (-0.17-0.08)	1.45 (1.29–1.64)	< 0.001*	1.35 (1.19–1.53)	< 0.001*
Control	1,036 / 69,418 (1.49)	1.49		1		1	
**Digestive disease**							
Osteoporosis	270 / 69,418 (0.39)	0.37	-0.13 (-0.20–0.06)	1.16 (0.93–1.46)	0.195	1.15 (0.91–1.44)	0.234
Control	344 / 69,418 (0.50)	0.49		1		1	
**Muscular disease**							
Osteoporosis	112 / 69,418 (0.16)	0.15	0.11 (0.08–0.14)	5.45 (3.05–9.73)	< 0.001*	5.38 (3.01–9.61)	< 0.001*
Control	30 / 69,418 (0.04)	0.04		1		1	
**Genitourinary disease**							
Osteoporosis	203 / 69,418 (0.29)	0.28	-0.11 (-0.17–0.05)	1.15 (0.88–1.49)	0.304	1.13 (0.87–1.47)	0.355
Control	270 / 69,418 (0.39)	0.39		1		1	
**Abnormal finding**							
Osteoporosis	688 / 69,418 (0.99)	0.93	-0.35 (-0.46–0.24)	1.14 (0.99–1.31)	0.073	1.07 (0.93–1.24)	0.349
Control	895 / 69,418 (1.29)	1.29		1		1	
**Trauma**							
Osteoporosis	633 / 69,418 (0.91)	0.86	-0.39 (-0.50–0.29)	1.06 (0.92–1.23)	0.419	1.08 (0.93–1.24)	0.319
Control	872 / 69,418 (1.26)	1.25		1		1	
**Others**							
Osteoporosis	79 / 69,418 (0.11)	0.11	-0.13 (-0.17–0.09)	0.69 (0.47–1.01)	0.059	0.71 (0.48–1.05)	0.083
Control	166 / 69,418 (0.24)	0.24		1		1	

Abbreviation: IR, incidence rate; IRD, incidence rate difference; SBP, systolic blood pressure; DBP, diastolic blood pressure; PY, person-year;

† Adjusted for age, sex, income, region of residence, SBP, DBP, fasting blood glucose, total cholesterol, obesity, smoking, alcohol consumption, and CCI scores

## Discussion

Our study investigated the association between osteoporosis under treatment and all-cause and specific-cause mortalities among 68,362 participants from a nationwide retrospective cohort in South Korea. Our findings suggested that osteoporosis under treatment was associated with a 41% reduced likelihood of all-cause mortality compared with the control group without osteoporosis, which was consistent across various demographic and clinical subgroups. Our analysis of specific-cause mortality revealed that mortality related to neoplasm and metabolic diseases was significantly lower in the osteoporosis group than in the control group.

The observed reduced all-cause mortality rate in osteoporosis under treatment is in accordance with previous observational evidence. The study by Li et al. that involved 87,935 participants from nationwide data of Taiwan indicated that osteoporosis medication was related to decreased all-cause mortality rate after hip (HR 0.75, 95% CI 0.73–0.77) and vertebral (HR 0.74, 95% CI 0.72–0.76) fractures [12]. Previous cohort studies also reported similar results, with their effect sizes varying from 0.25 to 0.92 [9–11, 13].

Various underlying mechanisms have been suggested to explain the observed association. First, osteoporosis medication may prevent the incidence of osteoporotic fractures. However, this alone does not fully account for the significant reduction in the mortality rate observed [10, 20]. A randomized controlled trial of zoledronic acid demonstrated that 8% of the total 28% reduction in mortality risk was attributable to a decreased risk of fractures [21]. Our analysis of the association between osteoporosis under treatment and specific-cause mortality also revealed significantly higher mortality due to muscular diseases, including fractures, compared with the control group without osteoporosis, which further supports the minor role of osteoporosis medication in preventing fractures. Second, osteoporosis medications have been related to a reduced risk of neoplasms, particularly breast cancer, leading to decreased mortality. Our findings, based on participants that mostly consisted of women (81.60%), also support this, showing that patients undergoing osteoporosis treatment had significantly lower mortality rates owing to neoplasms (HR 0.74, 95% CI 0.69–0.80). While only four observational studies were included, a meta-analysis suggested that bisphosphonate use was associated with reduced risk of any breast cancer (pooled relative risk [RR] 0.85, 95% CI 0.74–0.98) and invasive breast cancer (pooled RR 0.68, 95% CI 0.59–0.80) [22]. However, evidence from randomized controlled trials that investigated the effect of bisphosphonate on breast cancer have raised concerns regarding this observation: (1) Hue et al. reported non-significant results (alendronate: HR 1.24, 95% CI 0.84–1.83; zoledronic acid: HR 1.15, 95% CI 0.70–1.89) [23] and (2) a meta-analysis of individual participant data exhibited borderline significance (reduction in recurrence: RR 0.94, 95% CI 0.87–1.01; distant recurrence: RR 0.92, 95% CI 0.85–0.99; and mortality: RR 0.91, 0.83–0.99) [24]. Third, the reduction of bone loss and turnover associated with bisphosphonates may result in lower mortality rates among patients with osteoporosis under treatment. Both high bone loss and turnover are independent predictors of mortality [25, 26] as they can release toxins such as lead, which is linked to cognitive impairment, renal insufficiency, and increased mortality [27–29].

Notably, our study also observed reduced mortality due to metabolic diseases in patients with osteoporosis under treatment compared to the control group, showing an HR of 0.71 (95% CI 0.57–0.89) after adjustment. Our analysis for specific-cause mortality utilized the KCD (based on the ICD), of which the metabolic diseases (E00–E90) include diabetes mellitus and hyperparathyroidism. Interestingly, a meta-analysis including seven studies (two randomized trials and five observational studies) with a total of 1,233,844 participants found that bisphosphonate use was associated with a reduced risk of diabetes (RR 0.77, 95% CI 0.65–0.90). However, the subgroup analysis of randomized trials alone showed no significance (RR 0.93, 95% CI 0.74–1.18) [30]. Another meta-analysis reported that anti-resorptive agents might help mitigate hypercalcemia in primary hyperparathyroidism, based on evidence by decreased serum calcium levels (standardized mean difference: − 0.55, 95% CI − 0.94 to − 0.15) [31]. Since these results including our findings were primarily based on observational evidence and random-effect meta-analysis for the small number of studies could not guarantee sufficient statistical power to detect the accurate association, further randomized controlled trials are required to confirm these findings.

The findings of this study should be interpreted in consideration of some limitations. First, the investigated associations were based on an observational design, which cannot establish causality. The observed reduction in mortality rate among patients with osteoporosis under treatment should be understood not as the treatment being ‘directly’ effective in reducing mortality, but rather as the treatment being ‘associated’ with reduced mortality. Second, our study could not investigate the medication-specific associations since we included all patients with osteoporosis under treatment as a single group. However, this approach could benefit from a broader understanding of treatment effects, potentially highlighting overall trends. Third, while our analysis of specific-cause mortality provided further insights with 14 categories according to the KCD (based on the ICD), we could not present results for specific diseases of interest such as drug-specific adverse effects, pre-existing frailty, breast cancer, diabetes mellitus, and primary hyperparathyroidism. Future studies could focus on these specific diseases to provide a more detailed understanding of the treatment effects of different drugs on mortality in patients with osteoporosis. Fourth, a limitation of this study is the classification of all osteoporosis treatments as a single group, which prevents differentiation of effects among various drug classes, such as bisphosphonates, selective estrogen receptor modulators, and denosumab. Future studies should investigate drug-specific effects on mortality. Fifth, despite our efforts to minimize bias through propensity score weighting, unmeasured confounding factors such as dietary habits, physical activity levels, healthcare access, medication adherence, and socioeconomic disparities may have influenced our findings. These factors, which were not available in our dataset, could affect both osteoporosis treatment adherence and mortality outcomes.

## Conclusions

Despite these limitations, our nationwide retrospective cohort study involving 68,362 participants reinforced the observational evidence linking osteoporosis treatment with reduced all-cause mortality. Additionally, our analysis of specific-cause mortality suggested a potential mechanism for this observed reduced mortality, mediated by factors such as neoplasms and metabolic diseases such as diabetes mellitus and primary hyperparathyroidism. However, further studies, particularly randomized controlled trials, are required to confirm these results, establish causality, and explore the medication-specific effects on mortality. Clinically, these findings underscore the importance of osteoporosis treatment not only for bone health, but also for its potential broader impact on reducing mortality risk.

## Data Availability

Raw data for this study is not publicly available to preserve individuals’ privacy under the National Health Insurance Service, but are available from the corresponding author on reasonable request.

## Abbreviations

BMI: Body mass index
CCI: Charlson Comorbidity Index
CI: Confidence interval
HR: Hazard ratio
ICD: International Classification of Diseases
IRD: Incidence rate difference
KCD: Korean standard classification of diseases
SBP: Systolic blood pressure
DBP: Diastolic blood pressure
FBG: Fasting blood glucose
PS: Propensity score
RR: Relative risk
